# Molecular targeting of cell-permeable peptide inhibits pancreatic ductal adenocarcinoma cell proliferation

**DOI:** 10.18632/oncotarget.21939

**Published:** 2017-10-19

**Authors:** Shoki Sato, Toru Nakamura, Toyomasa Katagiri, Takahiro Tsuchikawa, Toshihiro Kushibiki, Kouji Hontani, Mizuna Takahashi, Kazuho Inoko, Hironobu Takano, Hirotake Abe, Shintaro Takeuchi, Masato Ono, Shota Kuwabara, Kazufumi Umemoto, Tomohiro Suzuki, Osamu Sato, Yusuke Nakamura, Satoshi Hirano

**Affiliations:** ^1^ Department of Gastroenterological Surgery II, Hokkaido University Graduate School of Medicine, Sapporo, Japan; ^2^ Division of Genome Medicine, Institute for Genome Research, Tokushima University, Tokushima, Japan; ^3^ Department of Medicine and Surgery, The University of Chicago, Chicago, IL, USA

**Keywords:** C16orf74, pancreatic ductal adenocarcinoma, cell-permeable peptide, molecular target therapy

## Abstract

**Background:**

*Chromosome 16 open reading frame 74* (*C16orf74*) is highly expressed in pancreatic ductal adenocarcinoma (PDAC) and is involved in cancer cell proliferation and invasion through binding to calcineurin (CN). Therefore, *C16orf74* is a good target for the development of a PDAC treatment. A cell-permeable dominant-negative (DN) peptide that can inhibit the C16orf74/CN interaction was designed to examine whether this peptide can inhibit PDAC cell proliferation *in vitro* and *in vivo*.

**Method:**

TheDN-C16orf74 peptide, which corresponds to the portion of C16orf74 that interacts with CN, was synthesized, and we assessed its anti-tumor activity in proliferation assays with human PDAC cells and the underlying molecular signaling pathway. Using an orthotopic xenograft model of PDAC, we treated mice intraperitoneally with phosphate-buffered saline (PBS), control peptide, or DN-C16orf74 and analyzed the tumor-suppressive effects.

**Result:**

DN-C16orf74 inhibited the binding of C16orf74 to CN in an immunoprecipitation assay. DN-C16orf74 suppressed PDAC cell proliferation, and the level of suppression depended on the expression levels of C16orf74 *in vitro*. DN-C16orf74 also exhibited anti-tumor effects in orthotopic xenograft model. Furthermore, the tumor-suppressive effect was associated with inhibition of the phosphorylation of Akt and mTOR.

**Conclusion:**

The cell-permeable peptide DN-C16orf74 has a strong anti-tumor effect against PDAC *in vitro* and *in vivo*.

## INTRODUCTION

Pancreatic ductal adenocarcinoma (PDAC) is one of the most aggressive of all malignancies [1, 2]. In 2017, it is estimated that there will be 53,670 new cases of pancreatic cancer in the USA , and PDAC is the fourth leading cause of cancer-related death [2]. Furthermore, in the USA, the number of patients with PDAC is expected to increase to 88,000 by 2030, up from 43,000 in 2010, which is projected to result in 63,000 deaths in 2030 compared to 36,888 in 2010 [1]. There are several reasons for PDAC's high mortality. First, because it is difficult to uncover early-stage lesions, most PDAC is diagnosed at an advanced stage, and many tumors are inoperable. Second, although many cytotoxic anti-cancer drugs, including gemcitabine (GEM), tegafur-gimestat-otastat potassium (TS-1), and 5-fluorouracil (5-FU), have been developed, the subsequent improvement in the 5-year survival rate for PDAC patients has been very modest [3, 4]. The FOLFIRINOX regimen (fluorouracil/leucovorin plus irinotecan plus oxaliplatin) has demonstrated a significant survival benefit compared with gemcitabine for patients with metastatic PDAC; however, only patients who have had a good performance status have been treated with FOLFIRINOX because of a higher toxicity than other regimens [5–7]. A recent report has shown that nab-paclitaxel (nab-PTX) plus GEM has controllable toxicity and superior efficacy than GEM alone as the first line treatment for patients with metastatic PDAC [8]. In this study, the median overall survival (OS) of the nab-paclitaxel plus gemcitabine group was significantly longer than that of the gemcitabine alone group (8.7 vs 6.6 months, hazard ratio [HR] = 0.72, *p* < 0.01) [8].

In addition, molecularly targeted therapies, such as receptor tyrosine kinase inhibitors, have been used to treat PDAC. However, gemcitabine plus erlotinib therapy failed to achieve a significant improvement (providing only a two-week extension in median OS over single-agent gemcitabine) [9]. Moreover, there is no evidence supporting the use of any other molecularly targeted therapies in PDAC. For these reasons, the development of a new anti-PDAC drug with high efficacy and no/minimal/low toxicity is urgently required.

The biological function of *Chromosome 16 open reading frame 74 (C16orf74),* which is located on *Chromosome 16q24.1*, is still not well known. However, we previously demonstrated that overexpression of the C16orf74 protein was an independent prognostic factor for patients with PDAC. We also showed that C16orf74 interacted with catalytic subunit alpha of protein phosphatase 3 (PPP3CA), which is an isozyme of calcineurin (CN), and that the interaction of these two proteins promoted PDAC cell proliferation and invasion [10]. In addition, we suggested that a CN-binding motif in C16orf74, PXIXIT, was critically important for PDAC proliferation. Hence, we hypothesized that disrupting the interaction between C16orf74 and CN might be a good approach to developing an effective treatment for PDAC.

In developing a new anti-cancer drug to inhibit the interaction of C16orf74 with CN, we designed a dominant-negative C16orf74 peptide with a cell-penetrating signal. Several studies have evaluated cell-penetrating peptides (CPPs) used for drug delivery *in vitro* and *in vivo*. These peptides function to inhibit molecular interactions in cancer cells and reduce cell growth [11, 12]. In particular, a polyarginine sequence (11R) is known as a signal for uptake of peptides or proteins into cultured cells, and this sequence is highly efficient for inducing uptake by mammalian cells [13, 14].

Here, we used the newly designed cell-permeable peptide to inhibit the C16orf74/CN interaction, and we evaluated whether this peptide could inhibit the growth of human PDAC cells *in vitro* and *in vivo* by using an orthotopic xenograft mouse model and elucidated the molecular mechanism of the growth-suppressive effect.

## RESULTS

### The design of the dominant-negative C16orf74 peptide

**Figure 1:**
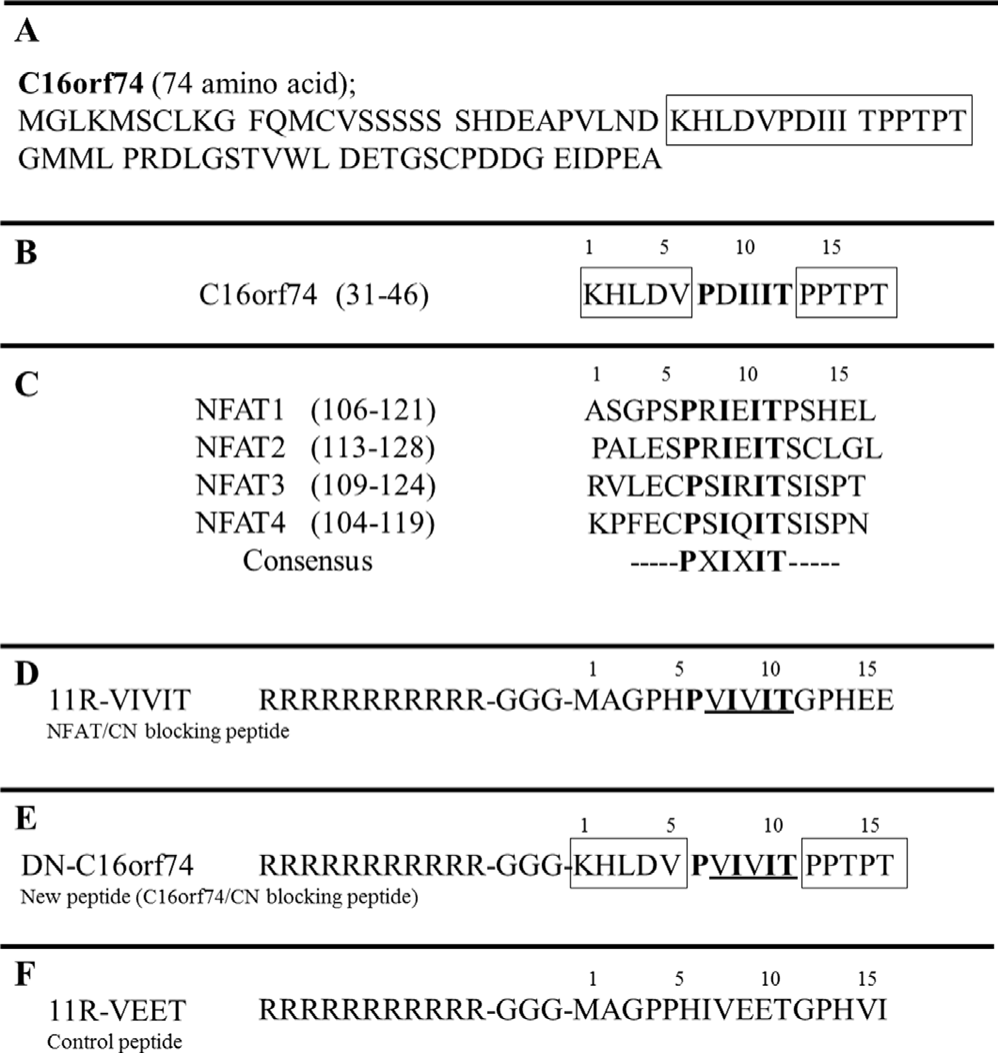
Peptide design to inhibit the binding of C16orf74 to calcineurin (CN) **(A)** The C16orf74 amino acid sequence is shown. **(B)** CN-binding sequences present in C16orf74. **(C)** CN-binding sequences present in the NFAT (nuclear factor of activated T cells) family of proteins. **(D)** The peptide sequence of 11R-VIVIT, a blocker of NFAT/CN binding, is shown. PVIVIT is the sequence that blocks the binding site. The 11-arginine (R) sequence facilitates the uptake of any peptide into cultured cells with high efficiency. **(E)** The sequence of DN-C16orf74 is shown. In this peptide, the C16orf74 sequence PDIIIT has been replaced with the sequence PVIVIT, which blocks the CN-binding site. **(F)** Control peptide; 11R-VEET sequences.

To design the dominant-negative C16orf74 peptide to inhibit the interaction between C16orf74 and CN, we analyzed the amino acid sequence encoded by C16orf74 (Figure 1A) and found a CN-binding consensus sequence, PXIXIT (Figure 1B, 1C). A previous report indicated that an 11R-VIVIT peptide that included the PVIVIT sequence inhibited the interaction between NFAT (nuclear factor of activated T cells) and CN (Figure 1C, 1D) [15, 16]. Hence, we synthesized the peptide 11R-GGG-KHLDVPVIVITPPTPT (DN-C16orf74; Figure 1E), in which the C16orf74 sequence PDIIIT was replaced with the sequence PVIVIT, as well as 11R-VEET as a control peptide (Figure 1F).

### The anti-tumor effect of DN-C16orf74 on PDAC cells

**Figure 2:**
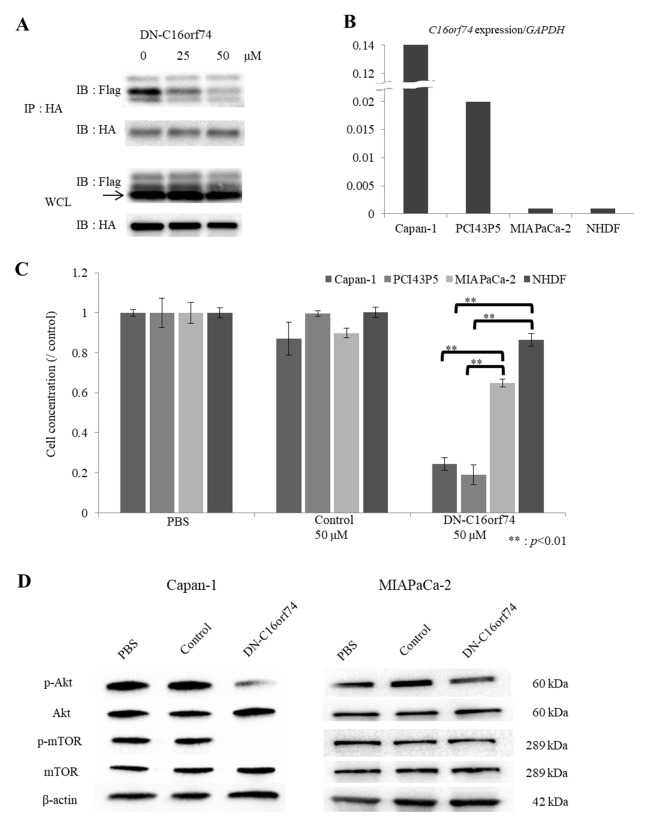
The anti-tumor effect of DN-C16orf74 *in vitro* **(A)** Lysates of co-transfected HEK293T cells (pCAGGS-C16orf74-3×Flag tag and pCAGGS-CN-HA tag) treated with DN-C16orf74 were immunoprecipitated with anti-HA antibody. Subsequently, western blotting analysis with anti-DDDDK (Flag) antibody detected C16orf74 and CN as co-immunoprecipitated proteins. Increasing the dose of DN-C16orf74 inhibited C16orf74/CN binding. The whole cell lysates showed equal expression of C16orf74 and CN in each treatment. IP: Immunoprecipitation, IB: Immunoblotting, WCL: Whole cell lysate. **(B)** The expression of *C16orf74* in PDAC cell lines and NHDFs is shown. Capan-1 cells had very high expression of *C16orf74*, and PCI43P5 cells had high expression of *C16orf74*; MIAPaCa-2 cells and NHDFs had low *C16orf74* expression. **(C)** The correlation between the anti-tumor effect of the DN-C16orf74 cell-permeable peptide and the *C16orf74* expression pattern in PDAC cell lines and NHDFs is shown. DN-C16orf74 inhibits cell proliferation of the Capan-1 and PCI43P5 cell lines, which have high expression of *C16orf74*, more than it inhibits the low-expression cell lines MIAPaCa-2 and NHDF. Control peptide does not inhibit cell proliferation. ^**^: *p* < 0.01. **(D)** To analyze molecular cellular signaling after treatment with DN-C16orf74, western blotting analyses of the expression of Akt, phospho (p)-Akt, mTOR, phospho (p)-mTOR, and β-actin in Capan-1 and MIAPaCa-2 cells treated with peptide were performed. In Capan-1, the expression of p-Akt and p-mTOR was decreased in response to treatment with DN-C16orf74, but their expression was maintained in response to treatment with the control peptide. In MIAPaCa-2, the expression levels were no change. The means and standard deviation (s.d.) of independent triplicate experiments in each group are shown.

To first examine the effect of DN-C16orf74 on the interaction between CN and C16orf74, we performed an immunoprecipitation assay. DN-C16orf74 inhibited the binding of C16orf74 to CN in HEK293T cells that had been co-transfected with a flag-tagged C16orf74 expression vector (pCAGGS-C16orf74-3×Flag tag) and an HA-tagged CN expression vector (pCAGGS-CN-HA tag), and the inhibitory effect was dependent on the dose of DN-C16orf74 (Figure 2A). These results suggested that DN-C16orf74 inhibits the C16orf74/CN interaction in a dose-dependent manner.

Subsequently, to examine the growth-suppressive effect of DN-C16orf74 on PDAC cell lines, we analyzed the expression levels of *C16orf74* in Capan-1, MIAPaCa-2, PCI43P5, and normal human dermal fibroblasts (NHDFs) at Real-time PCR, and found that Capan-1 and PCI43P5 cells expressed high levels of *C16orf74* but that MIAPaCa-2 cells and NHDFs exhibited low levels of expression (Figure 2B), similar to the results of a western blotting analysis reported previously [10]. As shown in Figure 2C, 50 μM DN-C16orf74 inhibited the proliferation of two cell lines with high *C16orf74* expression (Capan-1 and PCI43P5) but did not notably inhibit the proliferation of two cell lines with low *C16orf74* expression (MIAPaCa-2 cells and NHDFs) (*p* < 0.05). The control peptide did not exhibit a growth-suppressive effect. Reduction rates of DN-C16orf74/control were as shown below, Capan-1; 0.26, PCI43P5; 0.16, MIAPaCa-2; 0.78, and NHDF; 0.91, respectively.

Because C16orf74 contains the CN-binding consensus sequence present in NFAT proteins, we speculated that C16orf74 might activate proteins in cancer cells in a manner similar to NFATs. A previous report showed that knockdown of NFAT1 increased the phosphorylation levels of Akt and, conversely, overexpression of NFAT1 or NFAT4 decreased the phosphorylation levels of Akt in a colorectal cancer cell line [17]. Other reports have also shown a relationship between NFATs and Akt in several organs [18–20]. Furthermore, the Akt/mTOR pathway plays a key role in cancer cell proliferation [21, 22]. Therefore, we performed western blotting to examine the expression levels of Akt, phospho (p)-Akt, mTOR and phospho (p)-mTOR, in Capan-1, MIAPaCa-2, and NDHF cells treated with phosphate-buffered saline (PBS) or peptide (Control or DN-C16orf74) (Figure 2D and Supplementary Figure 1). In Capan-1 with high C16orf74 expression, the expression levels of p-Akt and p-mTOR decreased in response to treatment with DN-C16orf74, while no change was observed in cells treated with the control peptide or PBS. On the other hand, in MIAPaCa-2 and NHDF with low C16orf74 expression, no change of p-Akt and p-mTOR expression levels was observed (Figure 2D and Supplementary Figure 1)

### Anti-tumor effect of DN-C16orf74 peptide on human pancreatic cancer cells grown in the pancreas of nude mice

**Figure 3:**
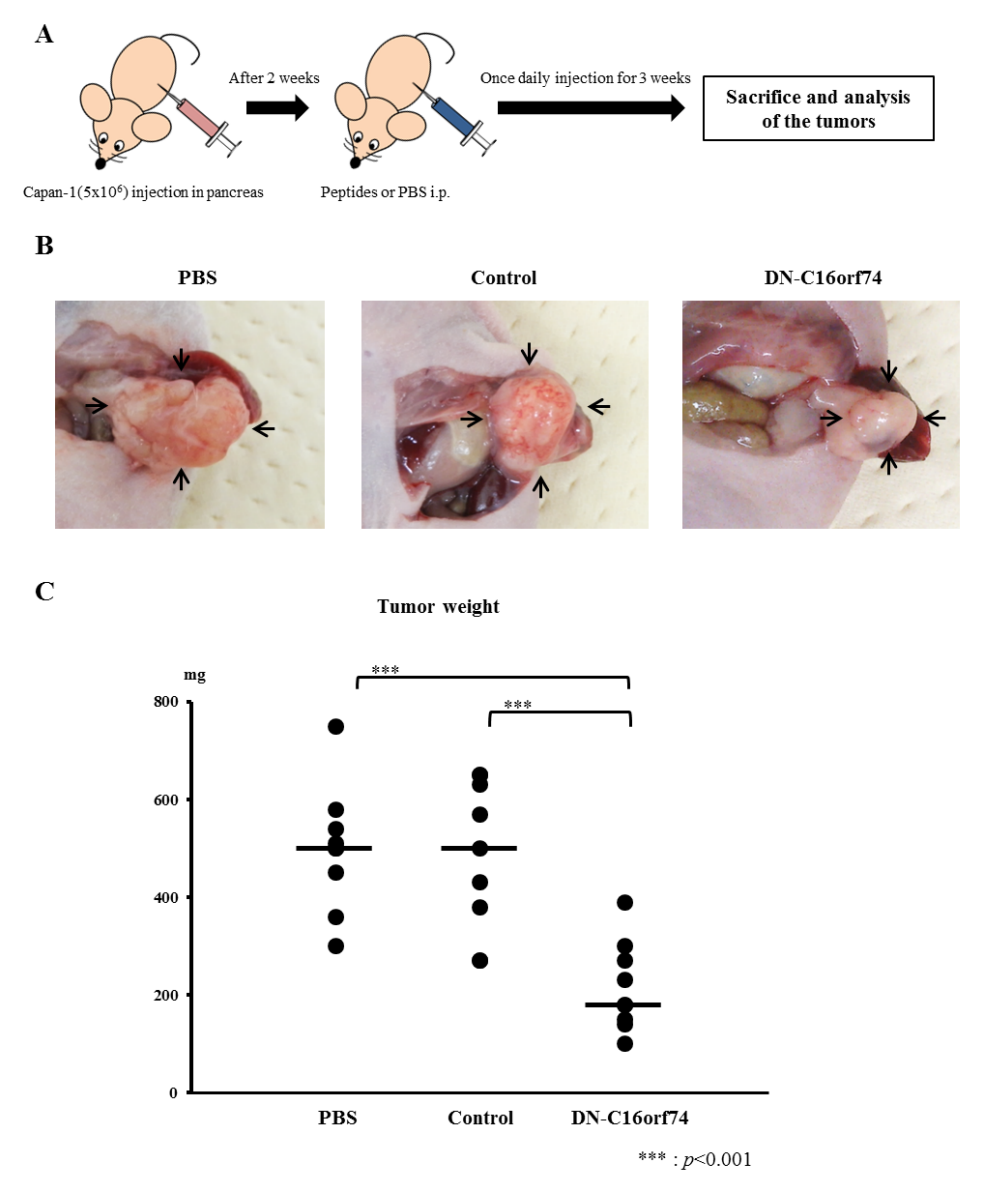
DN-C16orf74 treatment in an orthotopic xenograft model **(A)** The treatment protocol for the orthotopic xenograft model is shown. Capan-1 cells (5×10^6^) were implanted into the pancreas of BALB/c nu/nu mice. Two weeks later, the cell-permeable peptides (control peptide or DN-C16orf74, 9 mg/kg) or PBS were injected intraperitoneally once daily. Three weeks later, the mice were sacrificed. **(B)** Representative pancreatic tumors from the three groups (PBS, control, and DN-C16orf74) are shown. Arrowheads indicate pancreatic tumors. **(C)** Comparison of the tumor weights in the three groups. The dot plots (n = 9, respectively) represent tumor weight, and the bars represent the medians. Tumors treated with DN-C16orf74 were significantly smaller than the other tumors (vs. PBS; *p* < 0.001, and vs control; *p* < 0.001).

An orthotopic animal model was established with 27 nude mice in which human pancreatic cancer cells were implanted into the pancreas, and the mice were divided into three groups (treatment with PBS, control peptide, or DN-C16orf74, n = 9 in each group). For these orthotopic models, Capan-1 cells (high C16orf74 expression) were injected into the pancreas of nude mice [23]. Peptide treatment began 2 weeks later, when the tumors were considered to be established. After 3 weeks of treatment, all mice were euthanized and necropsied (Figure 3A, 3B). Tumor weights (median weight and range) were as follows: 500 mg (300-750), 500 mg (270-650), and 180 mg (100-390) in the PBS, control peptide and DN-C16orf74 groups, respectively (DN-C16orf74 vs PBS; *p* < 0.001, and DN-C16orf74 vs control peptide; *p* < 0.001, Figure 3C). The body weights of the mice were stable, and no side effect was observed in any groups. Body weight (median and range) after two weeks were follows: 20.6g (17.3-22.0), 21.6g (18.6-22.3), and 21.8g (19.4-22.5) in the PBS, control peptide and DN-C16orf74 groups, respectively. There was no significant difference.

### Immunohistochemical staining of Ki67, mTOR, p-mTOR, and TUNEL in orthotopic tumors

**Figure 4:**
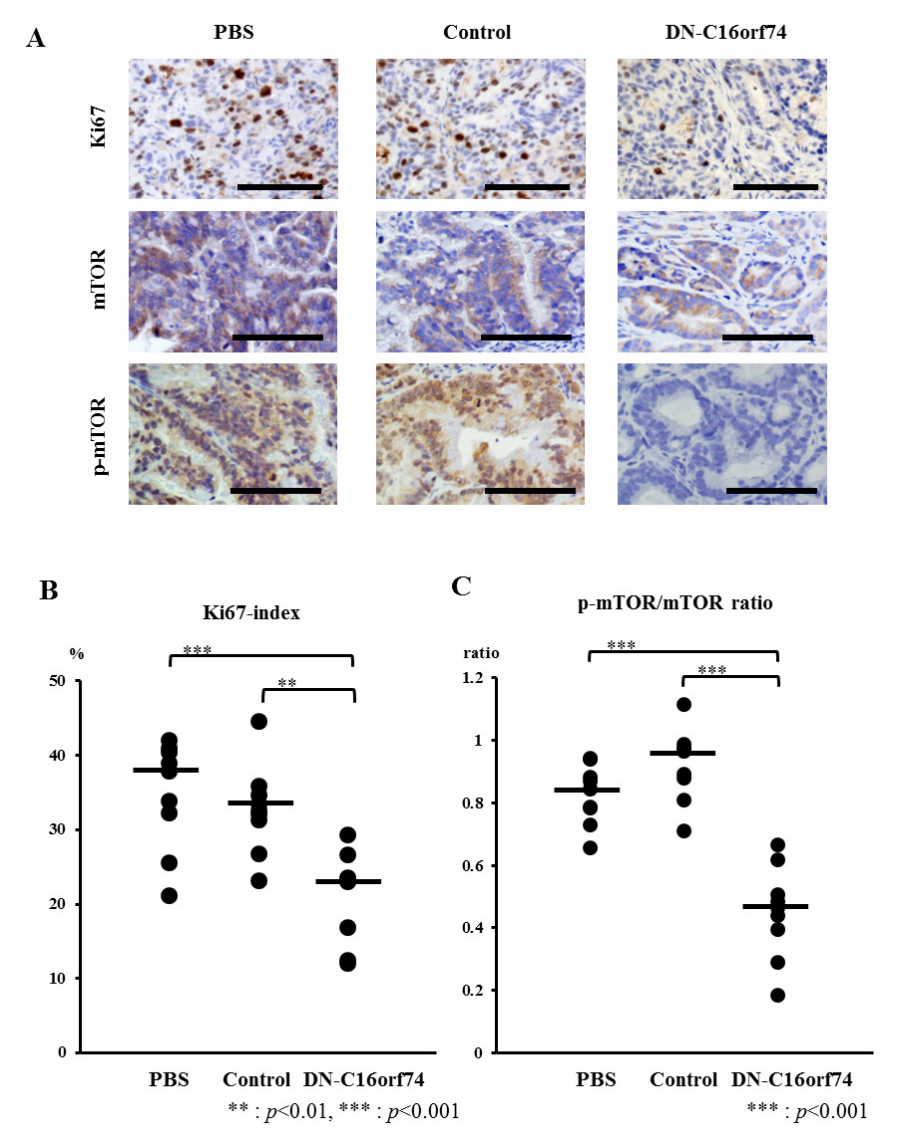
Analysis of cell proliferation (Ki67), mTOR, and p-mTOR in pancreatic tumors from peptide-treated mice **(A)** Immunohistochemical staining for Ki67, mTOR, and p-mTOR in pancreatic tumors treated with PBS, control peptide, or DN-C16orf74 is shown. Scale bars, 100 μm. **(B)** Comparison of the Ki67 index of the three groups. The Ki67 index is expressed as the percentage of Ki67-positive cells. The dot plots (n = 9, respectively) represent the Ki67 index, and the bars represent the medians. The Ki67 index is significantly lower in the DN-C16orf74 group than in the other groups (vs. PBS; *p* < 0.001, vs control; *p* < 0.01). **(C)** Comparison of the p-mTOR/mTOR expression ratio of the three groups. The dot plots (n = 9, respectively) represent the ratio, and the bars represent the medians. The ratio is significantly lower in the DN-C16orf74 group than in the other groups (vs. PBS; *p* < 0.001, vs control; *p* < 0.001).

To further characterize the effect of DN-C16orf74 in orthotopic tumors, we performed immunohistochemical staining for Ki67, mTOR, and p-mTOR (Figure 4A). Almost all of orthotopic tumors had C16orf74 expression (Supplementary Figure 2). The median Ki67-index was w37.9% (range, 21.1-42.0%) in the PBS group and 32.5% (range, 23.1-44.5%) in the control peptide group, but that in the DN-C16orf74 group was significantly lower, 23.3% (range, 12.1-29.3%) (vs the PBS group; *p* < 0.001, and vs the control peptide group; *p* < 0.01, Figure 4B). In addition, the median p-mTOR/mTOR ratio was 0.84 (range, 0.66-0.94) in the PBS group and 0.97 (range, 0.71-1.11) in the control peptide group, but that in the DN-C16orf74 group was significantly lower, 0.47 (range, 0.18-0.67) (vs the PBS group; *p* < 0.001, and vs the control peptide group; *p* < 0.001, Figure 4C). Furthermore, regression analysis showed significant correlation between orthotopic tumor weight and p-mTOR/mTOR ratio (r=0.701, p<0.001, Supplementary Figure 3). On the other hand, the median TUNEL numbers of the three groups were not significantly different respectively (Supplementary Figure 4).

## DISCUSSION

This study clearly indicates that the peptide DN-C16orf74, which corresponds to a portion of C16orf74, can inhibit the C16orf74/CN interaction in a dose-dependent manner and suppress PDAC cell proliferation *in vitro* and *in vivo*. Previous report showed that 46 PDAC cases (56.8%) had C16orf74 overexpression by immunohistochemical staining of a tissue microarray in 81 PDAC cases that underwent curative surgical resection, and these patients had poor prognosis rather than low expression patients [10]. This growth-suppressive effect was observed in PDAC cell lines showing high C16orf74 expression although the molecular mechanism by which the C16orf74/CN pathway can control cell growth is not clear. Because C16orf74 contains the consensus CN-binding sequence present in NFAT proteins, we hypothesized that C16orf74 might activate certain signaling pathways in cancer cells in a fashion similar to NFATs, and previous reports indicate a relationship between NFATs and Akt signaling [18–20]. Akt, a serine/threonine kinase, is a key regulator of cancer cell survival and proliferation and is an ideal target for the development of anti-cancer drugs [24, 25]. The phosphatidylinositol-3-kinase (PI3K)/Akt/mTOR pathway is also an important molecular signaling pathway in the regulation of tumor cell proliferation [26]. Therefore, we suspected that the C16orf74/CN interaction might be involved in the Akt/mTOR pathway and analyzed these molecules *in vitro* and *in vivo*. Our results indicate that DN-C16orf74 can inhibit C16orf74/CN binding *in vitro* and *in vivo*, and this resulted in suppression of the phosphorylation of Akt and mTOR, although further analysis will be necessary to determine the relationship between the C16orf74/CN interaction and the PI3K/Akt/mTOR pathway in the growth of PDAC.

The polyarginine sequence in DN-C16orf74 facilitates highly efficient nonspecific uptake of peptides into cells [14, 16]. Therefore, caution would be needed if DN-C16orf74 were used in the clinic because systemic peptide administration might not achieve a sufficient concentration in tumor tissue owing to uptake by normal vascular endothelial cells in blood vessels. Thus, cancer-specific peptide delivery systems are critical in considering the possibility of developing DN-C16orf74 for clinical use. In this regard, cancer cell-specific cell-permeable peptide signals, which can penetrate only the target cells *in vitro* and *in vivo* [27], might be applicable. If the polyarginine sequences in DN-C16orf74 peptide were modified to pancreatic cancer cell-specific penetrating sequences, a high-dose of DN-C16orf74 could be administered more effectively and safely. In addition, peptide stability in the blood is an important issue. For example, the peptide drug Calperitide (α-human A-type natriuretic peptide) is used in the clinic, but since the half-life of this peptide is very short [28], therefore, DN-C16orf74 peptide has been modified to make it more stable in the blood. The other possibility for clinical use of DN-C16orf74 is direct injection of the peptide into the pancreatic tumor via endoscopic ultrasound (EUS)-guided fine needle injection, which has been attempted for various agents, including oncolytic virus, mixed lymphocyte culture, and immature dendritic cells [29, 30]. In addition, intraperitoneal administration of this peptide may be considered for pancreatic cancer patients with peritoneal dissemination. Recently, a phase II clinical trial of intraperitoneal paclitaxel for PDAC patients with peritoneal metastasis was reported [31]. This report showed that the median survival time for treated patients was 16.3 (11.47-22.57) months, and approximately 30% of the patients underwent conversion surgery after control of the peritoneal dissemination.

In conclusion, DN-C16orf74 inhibited the C16orf74/CN interaction in a dose-dependent manner and suppressed PDAC proliferation *in vitro* and *in vivo* through reduction of Akt and mTOR phosphorylation. C16orf74 could be an ideal molecular target for PDAC treatment, and DN-C16orf74 is a potential drug candidate for use as a C16orf74 inhibitor.

## MATERIALS AND METHODS

### Peptide design and synthesis

Three peptides were synthesized by Sigma Life Science (Tokyo, Japan). The amino acid sequences of the peptides are shown below.

DN-C16orf74; RRRRRRRRRRR-GGG-KHLD VPVIVIPPTPT

11R-VIVIT; RRRRRRRRRRR-GGG-MAGP HPVIVITGPHEE

11R-VEET; RRRRRRRRRRR-GGG-MAGP PHIVEETGPHVI

The peptide 11R-VEET is non-functional and was used as the control peptide [16]. The peptides were purified by preparative reversed-phase HPLC and were >90.5% pure, and had the expected amino acid compositions and mass spectra.

### Cell lines

The human PDAC cell lines Capan-1 and MIAPaCa-2 and the embryonic kidney cell line HEK293T were purchased from the American Type Culture Collection (ATCC, Rockville, MD, USA). NHDFs were purchased from TAKARA BIO (Shiga, Japan). The PCI43P5 cell line was previously established from surgically resected PDAC tissues at our institute [32]. All cell lines were cultured in the appropriate medium as directed by the manufacturers or according to published studies. Capan-1 was cultured in IMDM (WAKO, Tokyo, Japan) with 20% fetal bovine serum (Cell Culture Bioscience), and 1% penicillin/streptomycin (Life Technologies), and MIAPaCa-2, PCI43P5, NHDF, and HEK293T cells were cultured in RPMI 1640 (WAKO) with 10% fetal bovine serum, and 1% penicillin/streptomycin. All cells were incubated at 37°C in a mixture of 5% CO_2_ and 95% air.

### Real-time PCR

Total RNA was extracted with an RNAeasy Mini Kit (Qiagen), and cDNA was synthesized with PrimeScript RT Master Mix (TAKARA BIO). Real-time PCR was performed with Fast SYBR ® Green Master Mix (Life Technologies) and specific primers (Supplementary Table 1), and the products were analyzed using StepOne (Applied Biosystems). Each peak in the melting curve was validated for primer specificity. All experiments were performed in duplicate for each sample. Gene expression was normalized to *GAPDH* expression, and fold expression changes were calculated using the ΔΔCt method. A list of the primers is provided in Supplementary Table 1.

### Western blotting and immunoprecipitation

Total cell lysate protein samples were collected with RIPA buffer in the presence of the protease inhibitors aprotinin (WAKO) and phenylmethylsulfonyl fluoride (PMSF, WAKO). Recombinant C16orf74 protein was produced in our laboratory. Samples were resolved using 15% SDS-PAGE and then transferred to PVDF membranes (Bio-RAD, Tokyo, Japan). Membranes were probed with primary antibodies against the targets followed by goat anti-mouse or rabbit IgG as the secondary antibodies (1:10,000 dilution). Primary antibodies and dilutions are shown in Supplementary Table 2, and secondary antibodies were purchased from Jackson ImmunoResearch. Immunoreactivity was detected with an enhanced chemiluminescence detection system (GE Healthcare). Equal loading was confirmed with β-actin. A list of the antibodies is presented in Supplementary Table 2.

For the immunoprecipitation assay, co-transfected HEK293T cells (pCAGGS-C16orf74-3×Flag tag and pCAGGS-CN-HA tag) were treated with DN-C16orf74 peptide (0, 25, or 50 μM) for 12-24 hours and lysed with RIPA buffer [10]. Immunoprecipitation was performed with mouse anti-HA antibody (Medical and Biological Laboratories, Nagoya, Japan). The antibodies were removed by incubation with Protein A-Agarose (sc-2001; Santa Cruz Biotechnology, Santa Cruz, CA), and the wash step was repeated 5 times. Proteins were extracted with SDS sample buffer and separated by 10-20% gradient SDS-PAGE (Bio-Rad). To examine the interaction between Flag-C16orf74 and HA-CN, we analyzed the immune complexes using western blotting with mouse anti-DDDDK(Flag)-tag antibody (Medical and Biological Laboratories) and anti-HA antibody.

### Cell proliferation assay (WST assay)

Cell proliferation was assessed after treatment using Cell Counting Kit-8 (WST-8, DOJINDO, Kumamoto, Japan). Cell lines were used in this assay; 5×10^3^ cells were seeded in 96-well plates and cultured for 24 hours before treatment. Cells were treated with control peptide or DN-C16orf74 (50 μM) or PBS as the control for 24 hours. At 4 hours after WST-8 application, absorbance was measured at 450 nm using a microplate reader. The viability of cells treated with PBS was set at 100%, and the absorbance of wells with medium but without cells was set to zero.

### Treatment of the cells with peptides to analyze molecular signaling

To analyze how molecular signaling in PDAC cells was affected by DN-C16orf74, total cell lysate protein samples were collected from Capan-1, MIAPaCa-2 and NHDF cells treated with each of two peptides. The cells were treated for 6 hours with 25 μM peptide (a control peptide or DN-C16orf74) or PBS as a control, and protein samples were collected. Western blotting analyses of Akt, p-Akt, mTOR, p-mTOR, and β-actin were performed.

### Immunohistochemical staining

Immunohistochemical staining of xenograft tumors was performed as previously described [33]. All samples were formalin-fixed and paraffin-embedded. Primary antibodies, dilutions, and antigen retrieval methods are shown in Supplementary Table 2. Negative controls were established by not adding primary antibody. All specimens were evaluated by two investigators (S.S. and H.T.). For Ki67 expression, staining was considered positive when nuclear staining was observed. The Ki67-index is expressed as the percentage of Ki67-positive cells in three independent high-power (200×) microscopic fields for each tissue sample. For mTOR and p-mTOR expression, staining was considered positive when cytoplasmic staining was observed. Analysis of the p-mTOR/mTOR ratio was performed in three independent high-power microscopic fields for each tissue sample. For apoptosis analysis, a TUNEL assay was performed using a commercially available TUNEL kit, the Apoptosis *in situ* Detection Kit *Wako* (WAKO). A list of the antibodies is presented in Supplementary Table 2.

### Animals and implantation of tumor cells for orthotopic xenografts

Capan-1 cells were harvested from subconfluent cultures by brief exposure to 0.25% trypsin. Trypsinization was stopped with medium containing 10% FBS, and cells were resuspended in Hank's balanced salts solution (HBSS, Life Technologies). For mouse studies, female BALB/c-nu/nu mice were purchased from CLEA, Japan. At the age of 6-8 weeks, mice were anesthetized, and a left subcostal incision was made. Capan-1 cells were resuspended (5×10^6^/100 μl HBSS) and injected subcapsularly into a region of the pancreas just beneath the spleen through the subcostal incision. A 27-gauge needle and a 1-mL disposable syringe were used. A successful subcapsular intrapancreatic injection of tumor cells was identified by the appearance of a fluid bleb without intraperitoneal leakage. To prevent such leakage, a cotton swab was held for 30 seconds over the injection site. One layer of the abdominal wound was closed with wound clips (Auto-clip; Clay Adams, Parsippany, NJ) [34]. The animals tolerated the surgical procedure well, and no anesthesia-related deaths occurred. Animal procedures were conducted according to the guidelines of the Hokkaido University Institutional Animal Care and Use Committee using an approved protocol.

### Peptide treatment protocol for orthotopic xenograft model

Starting two weeks after implantation of Capan-1 cells, the mice were intraperitoneally injected with DN-C16orf74 peptide or control peptide (9 mg/kg, 0.5 ml) (using a 27-gauge needle and a 1-mL disposable syringe) once daily for 3 weeks. As a negative control, the same volume of PBS was used. The mice were sacrificed under anesthesia approximately 24 hours after the final injection of peptide or PBS, and the pancreatic tumors were resected. After the tumors were weighed (mg), they were fixed in 10% formalin and embedded in paraffin wax for immunohistochemical staining procedures.

### Statistical analysis

All analyses were performed using StatFlex 6.0 software (Artech Co., Osaka, Japan). Student’s *t-*test, a chi-square test, and Fisher’s exact test were used for comparisons, as appropriate. The relationship between orthotopic tumor weight and p-mTOR/mTOR ratio was evaluated using the coefficient of determination (r) in the goodness of fit model. Results were considered significant at *p* < 0.05.

## SUPPLEMENTARY MATERIALS FIGURES AND TABLES


