# Supplementary material for: Molecular targeting of cell-permeable peptide inhibits pancreatic ductal adenocarcinoma cell proliferation
Source: Oncotarget. 2017 Oct 19;8(69):113662–72. doi: 10.18632/oncotarget.21939 (PMC5768354; doi:10.18632/oncotarget.21939)
Supplement: Supplementary file 1 [file oncotarget-08-113662-s001.pdf]

Molecular targeting of cell-permeable peptide inhibits pancreatic ductal adenocarcinoma cell proliferation

SUPPLEMENTARY MATERIALS

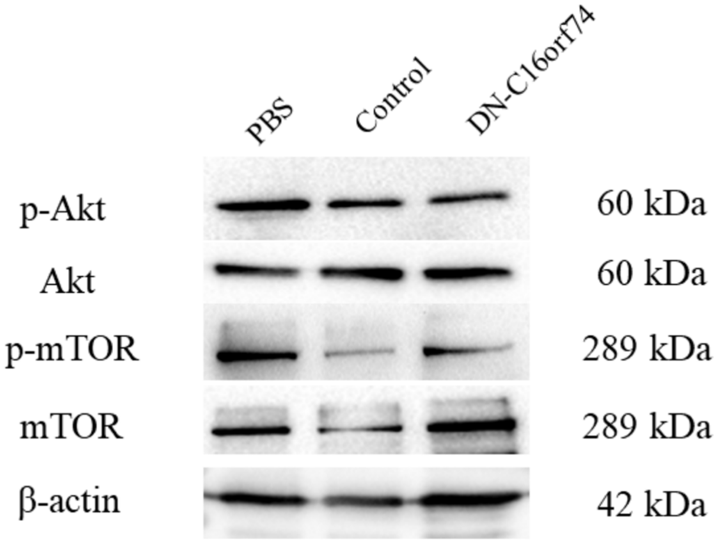

Supplementary Figure 1: To analyze molecular cellular signaling after treatment with DN-C16orf74, western blotting analyses of the expression of Akt, phospho (p)-Akt, mTOR, phospho (p)-mTOR, and β-actin in NHDF cells treated with PBS or peptide were performed. The expression levels of p-Akt and p-mTOR were no change.

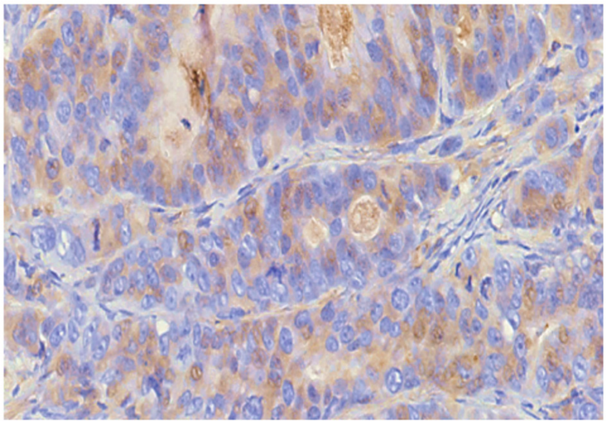

Supplementary Figure 2: C16orf74 expression in orthotopic xenograft mouse model tumors (Capan-1 cells) by immunohistochemical staining. Almost all of orthotopic tumor cells had C16orf74 expression.

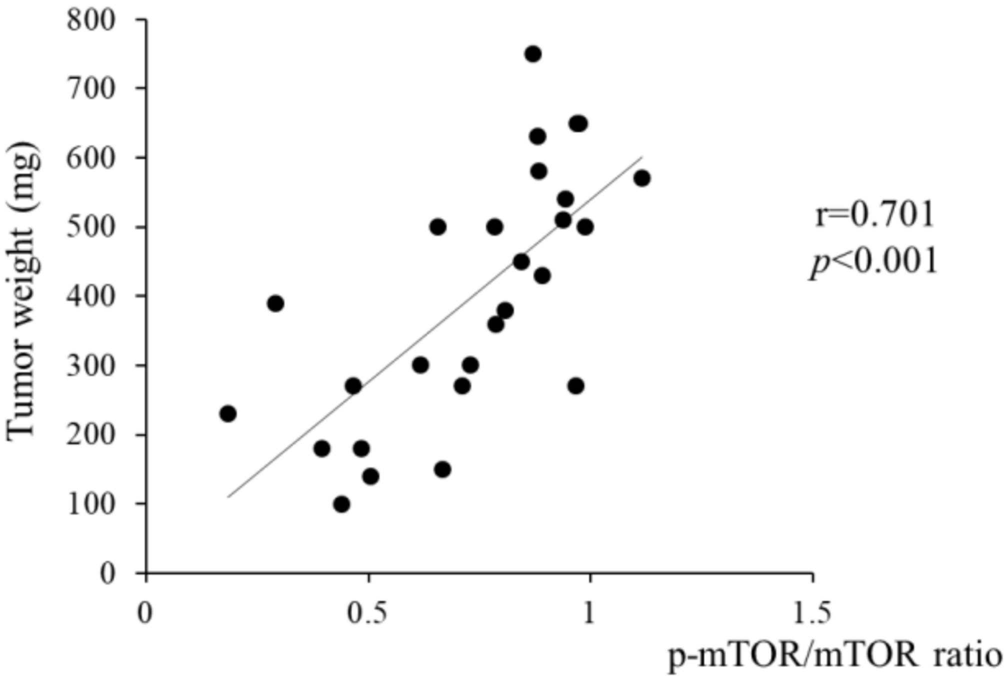

Supplementary Figure 3: Regression analysis of correlation between orthotopic tumor weight and p-mTOR/mTOR ratio. There was significant correlation between them (r=0.701, p<0.001).

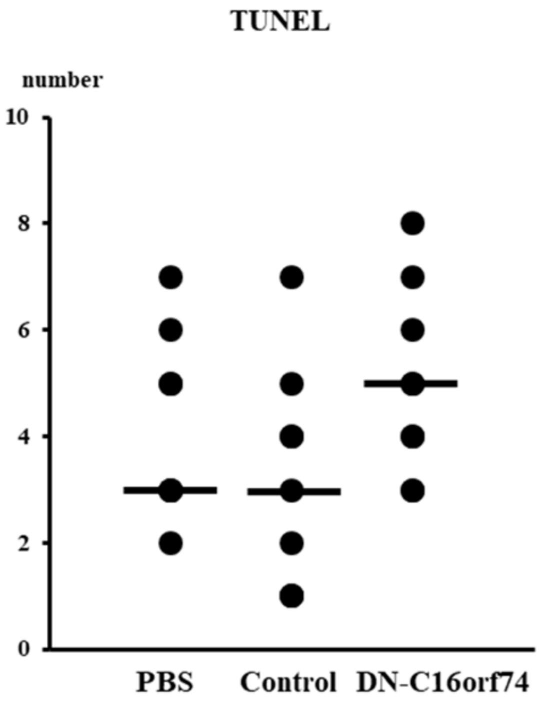

Supplementary Figure 4: The median TUNEL numbers (range) of the three groups were not significantly different: PBS 3 (2-7) vs. control peptide 3 (1-7) vs. C16orf74 5 (3-8), respectively.

Supplementary Table 1: List of primers for PCR

Gene	Forward	Reverse
C16orf74	5'-GAGGGACTTGGGGAGCAC-3'	5'-TCTGGGTCGATTTCTCCATC-3'
GAPDH	5'-GAGAGGCCCTATCCCAACTC-3'	5'-GCGAACTTTATTGATGGTATTCAA-3'

Supplementary Table 2: Lists of antibodies

**Western blotting**					
**Molecule**		**Clone**	**Dilution**		**Manufacture**
Akt	pRb		1:1000		Cell signaling
Phospho-Akt (Ser473)	mRb	D9E	1:2000		Cell signaling
mTOR	mRb	D9C2	1:1000		Cell signaling
Phospho-mTOR (Ser2448)	mRb	7C10	1:1000		Cell signaling
β-Actin	mMo	C4	1:1000		Millipore
Immunohistochemistry					
**Molecule**		**Clone**	**Dilution**	**Retrieval**	**Manufacture**
Ki67	mMo	MIB-1	1:300	EDTA pH9.0, 20min	Dako
mTOR	mRb	D9C2	1:100	Citric acid pH 6.0, 10min	Cell signaling
Phospho-mTOR (Ser2448)	mRb	49F9	1:100	Citric acid pH 6.0, 10min	Cell signaling

mRb : monoclonal rabbit, mMo : monoclonal mouse
